# *Candida auris* screening of high-risk patients: a descriptive comparison of 2 strategies

**DOI:** 10.1017/ash.2024.504

**Published:** 2025-01-27

**Authors:** Laura Pedersen, Aldo Barajas-Ochoa, Kaila Cooper, Jenna Price, Kathryn Hannum, Yvette Major, Patrick R. Ching, Barry Rittmann, Alexandra Bryson, Christoper Doern, Michelle Doll

**Affiliations:** 1 Division of Infectious Diseases, Department of Medicine, Virginia Commonwealth University, Richmond, VA, USA; 2 Healthcare Infection Prevention Program, Virginia Commonwealth University Health System, Richmond, VA, USA; 3 Division of Microbiology, Department of Pathology, Virginia Commonwealth University, Richmond, VA, USA

## Abstract

We compared two *Candida auris* screening strategies in high-risk patients.

The positivity rates for point prevalence survey (PPS) and admission screening were similar: 3.9% versus 3.4%, respectively, *P* = 1.00. Approximately 3% of high-risk patients are colonized, thus there is a need for a universal infection prevention approach for *C. auris.*

## Introduction

*Candida auris* was first isolated from clinical culture in 2009 and then in the bloodstream in 2011. This fungus can persist in the environment and form environmental reservoirs.^1,2^ *C. auris* bloodstream and other deep-seeded infections carry a high risk of mortality.^3^

Here we describe two screening strategies for this emerging multidrug-resistant pathogen. Our facility is an 865-bed tertiary care hospital. *C. auris* was first identified in an outbreak in our hospital in early 2022. The index patient for this cluster was transferred from an outside hospital in December 2021. Subsequently, we experienced ongoing outbreaks in the setting of a known regional outbreak in Virginia. From the initial outbreak, before the data collected in this study, we had evidence that two unique isolates were present based on whole genome sequencing, suggestive of both internal transmission and outside importation. We were also informed by the Virginia Health Department of outbreaks in long-term care facilities that feed our facility. We performed point prevalence surveys (PPSs) on all patients on a unit when a new case of *C. auris* was identified (i.e. in clinical cultures) per CDC recommendation. Given the frequency of new *C. auris* cases identified, and the frequency of subsequent PPS, we instituted admission screening. There was clinical suspicion that *C. auris* was being transmitted into our facility via admissions from post-acute care facilities. Thus, we designed and implemented admissions screening beginning August 2023, using a protocol that expanded on prior CDC recommendations.^4^

## Methods

**Figure 1.**
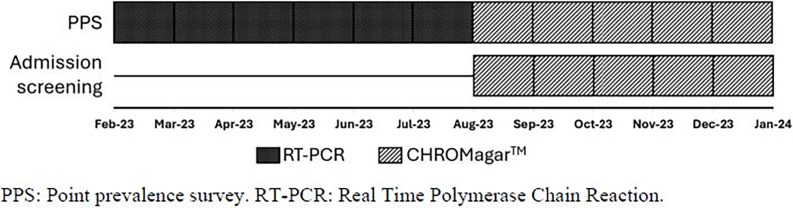
Chronological depiction of the screening strategies and testing used.

Initially, point prevalence testing was performed via real-time PCR (RT-pcr) with the assistance of the Virginia Department of Health from 2/2023 to 8/2023. Collaborating with the Microbiology Division, we developed and validated a *C. auris* screening assay at our institution utilizing CHROMagar^TM^, a *Candida spp.* selective media.^5^ This in-house screening test was launched on 8/2023, resulting in all PPS and admission samples being processed via selective media after 8/2023 (Fig. 1). PPS was indicated if a patient had a new positive clinical culture on the unit and had been admitted >48 hours. Tested patients were on general medical and surgical wards, medical and surgical intensive care units, and the burn unit, which is a combination of general level and intensive care. Weekly PPS was continued on all patients on the affected unit until the samples for two consecutive weeks were all negative. The approximate turnaround for a finalized result using our in-house assay was 3 days.

Patients were considered high risk on admission if they were admitted from “anywhere that was not home,” meaning any congregate living, jail/prison, or post-acute care facility. Collectively, we considered these “post-acute care facilities” (PACs). We started screening admissions from PACs on 8/2023 using our in-house assay. A scripted message explaining that the Infection Prevention nursing team would be placing and following up on the screening order, and other basic information was sent via secure chat in the electronic medical record to the admitting attending physician, first pager on call, and the bedside nurse. Specimens were collected via a swab of the bilateral axilla and groin. At the time of manuscript submission, we continue to screen high-risk admissions and conduct PPS as described above.

We provide descriptive statistics and compared the overall positivity rates of exposure to PPS versus PAC admission screenings using Fisher’s Exact Test with statistical significance set at *P* < 0.05. All statistical analysis was performed in SAS 9.4 software (Copyright © 2013, SAS Institute Inc.)

## Results

Point prevalence screening was performed on affected units from February 2023 onward, and data presented in this report was collected through December 2023. A total of 533 tests on 367 patients were performed. Twenty-one were positive (3.9% positivity rate). Three additional samples were either unable to be processed or indeterminate. Sixty-eight patients had repeat testing weekly for ≥2 weeks. Most remained negative, but 5 tested positive after variable amounts of negative-week intervals: 3 patients at week 2, 1 at week 4, and 1 at week 5.

Screening strategy two was implemented in August 2023, and data was collected through December 2023. A total of 89 patients were tested, with 3 positive results (3.4% positivity rate). Each positive patient was from a unique facility.

Four patients refused PPS. There were no known patient refusals for admission screening.

The overall positivity rates for PPS and admission screening indications were not significantly different: 21/533 (3.9%) completed PPS tests versus 3/86 (3.4%) admission screens, *P* = 1.00. When considering the interval 8/2023-12/2023 when the in-house assay was used for both PPS and admission screening, positivity rates remained the same: PPS 8/220 (3.5%), versus admission screening 3.4%, *P* = 1.00. Therefore, we had 8 new episodes of within-facility transmission after admission screening began.

## Discussion

We compared two screening strategies for C*. auris* at a single academic medical center. Similar positivity rates, 3.4% and 3.9%, were found for admissions from regional facilities and point prevalence strategies. Our positivity rates were similar to those found by Rosa and colleagues who reported a *C. auris* positivity rate of 3.23% (2.40%-4.43%) after implementing rapid diagnostic RT-PCR for admission screening.^6^ These data raise the concern that there may be a baseline positivity rate of *C. auris* colonization within the acute and post-acute care facilities in our region and beyond, despite heightened attention to and validation of core infection prevention practices. With 3% of high-risk patients colonized, there is a need for a more universal approach to *C. auris* infection control, as the current recommendations that require active screening and isolation are labor intensive, healthcare resource intensive, and typically identify patients too late. Novel approaches to control the spread of *C. auris* are urgently needed, such as leveraging topical agents to decrease/combat chronic patient colonization.^7^

A strength of this study is the novelty of comparing admission screening strategies to point-prevalence screening. We began universal admission screening for every patient not admitted from home. Therefore, our admission screening expanded beyond the CDC recommendation for high-risk patients or facilities.^4^ One barrier to using a “high risk facility” approach to screening was that we were not privy to information on *which* PACs were having outbreaks in our region, but only that outbreaks were occurring. For this reason, we also continued admission screening as a standing procedure, regardless of whether an outbreak work up was in process internally. It is plausible that other facilities were conducting their own universal admission screenings, but we did not find published experiences of this approach outside of published outbreaks. This intervention also shows the value of interdisciplinary collaboration between bedside nursing for collecting samples, the microbiology division for assay development, and the healthcare infection prevention team in planning and executing the screening program.

We could not systematically compare the two screening methods used in this study because we used the default method available. Therefore, comparing the in-house assay and RT-PCR is beyond the scope of this investigation. Nevertheless, both methods have been shown to be cost-effective and have similar efficacy in the literature.^8^

A limitation of our study is not having culture isolates of the PCR-positive cases. Not having whole genome sequencing available restricts opportunities for contact tracing. Nevertheless, it still provides valuable surveillance data, as clade does not impact the use of precautions for *C. auris.* It should also be noted that our university did not place patients on preemptive precautions. To do this would have been prohibitive for patient flow in our large, tertiary, safety-net hospital. An additional limitation is the potential difference in clinical characteristics of patients screened. Patients admitted from post-acute care facilities or other hospitals are not equivocal to patients already hospitalized in general wards and intensive care units.

We employed two different *C. auris* screening strategies at our hospital in the setting of known outbreaks in post-acute care facilities in the area. Facilities experiencing *C. auris* acquisition, in the form of infection or colonization, should consider screening admissions, especially if surrounding acute care facilities are experiencing outbreaks or evidence of ongoing acquisition despite high fidelity of infection prevention practices. Enhanced cleaning and de-colonization strategies will be particularly important given the persistence of this organism in these reservoirs, which may decrease the efficacy of contact isolation as a primary control strategy. Additional remediation strategies are urgently needed as *C. auris* becomes increasingly common in healthcare facilities despite efforts to identify and isolate affected patients.
